# Efficient expression of heterologous genes by the introduction of the endogenous glyceraldehyde-3-phosphate dehydrogenase gene intron 1 in *Ganoderma lucidum*

**DOI:** 10.1186/s12934-021-01654-8

**Published:** 2021-08-21

**Authors:** Hao You, Bin Sun, Na Li, Jun-Wei Xu

**Affiliations:** 1 Faculty of Life Science and Technology, Kunming University of Science and Technology, Kunming, 650500 China; 2 Faculty of Science, Kunming University of Science and Technology, Kunming, 650500 China

**Keywords:** *Ganoderma*, Expression of heterologous genes, Intron, mRNA accumulation, Protein expression, Enzyme activity

## Abstract

**Background:**

*Ganoderma lucidum*, a well-known medicinal mushroom, has received wide attention as a promising cell factory for producing bioactive compounds. However, efficient expression of heterologous genes remains a major challenge in *Ganoderma*, hindering metabolic regulation research and molecular breeding of this species.

**Results:**

We show that the presence of glyceraldehyde-3-phosphate dehydrogenase gene (*gpd*) intron 1 at the 5′ end of, the 3′ end of, or within the heterologous phosphinothricin-resistant gene (*bar*) is efficient for its expression in *G. lucidum*. The enhanced expression of *bar* is exhibited by the higher accumulation of mRNA and increased amounts of protein. Moreover, the insertion of the *gpd* intron 1 in the β-glucuronidase gene (*gus*) elevates its mRNA accumulation and enzyme activity, which facilitates the use of this reporter gene in *Ganoderma*.

**Conclusions:**

This study has demonstrated the importance of the introduction of *gpd* intron 1 for the efficient expression of *bar* and *gus* in *G. lucidum*. The presence of the *gpd* intron 1 in heterologous genes increases levels of mRNA accumulation and protein expression in basidiomycete *Ganoderma*. The developed method may be utilized in upregulating the expression of other heterologous genes in *Ganoderma*.

**Supplementary Information:**

The online version contains supplementary material available at 10.1186/s12934-021-01654-8.

## Background

*Ganoderma lucidum*, a well-known medicinal mushroom, can synthesize a variety of bioactive products such as ganoderic acids, ganoderols, polysaccharides, immunomodulatory proteins, nucleotides, and sterols [1]. It has received wide attention as a promising cell factory for producing these valuable compounds in recent years [2–7]. The genome, transcriptome, and proteome of *G. lucidum* have been sequenced [8–10]. *G. lucidum* has been suggested as a model species for studying the biology of basidiomycetes and biosynthesis of secondary metabolites [8, 11].

Molecular genetic approaches tools such as genetic transformation, disruption, and deletion of target genes have been developed in *G. lucidum* [12–16]. However, efficient expression of heterologous genes remains a main challenge in *Ganoderma*, although it would be valuable for metabolic regulation and molecular breeding. Successful expression of heterologous genes in basidiomycetes may depend on several factors, including an effective promoter, codon optimization, and the presence of introns [12, 17, 18]. Among those elements, introns have a significant effect on the expression of heterologous genes. In some mushroom species such as *Clitopilus passeckerianus*, *Coprinus cinereus*, and *Schizophyllum commune*, the addition of an intron is required for the efficient expression of the green fluorescent protein gene (*gfp*), the hygromycin-resistant gene, and the phleomycin-resistant gene [19–21].

Intron-containing *G. lucidum* genes represent 85.4% of predicted genes (approximately 16,113) in its 43.3-Mb genome [8], indicating the importance of introns in *Ganoderma*. In a previous study, we found that an extra fragment, including *gpd* exon 1, intron 1, and 3-bp exon 2 at the 5′ end of *gfp* and phosphinothricin-resistant gene (*bar*), was essential for their expression in *G. lucudium* [16]. However, the role of *gpd* intron 1 in regulating the expression of heterologous genes has not been yet thoroughly investigated in *G. lucidum*. Furthermore, how *gpd* intron 1 affects gene transcription, protein expression, and enzyme activity when heterologous genes are expressed in *G. lucidum* remains unclear.

In this study, we show that the presence of *gpd* intron 1 at different locations is effective in enhancing the expression of heterologous *bar* in *G. lucidum*. The efficient expression of heterologous genes is due to higher accumulation of mRNA and increased amount of protein. Moreover, the insertion of *gpd* intron 1 in the *gus* enhances its mRNA and enzyme activity, which facilitates the use of this reporter gene in *Ganoderma*.

## Results and discussion

### Endogenous *gpd* intron 1 increases the expression of the heterologous *bar* in *G. lucidum*

**Fig. 1:**
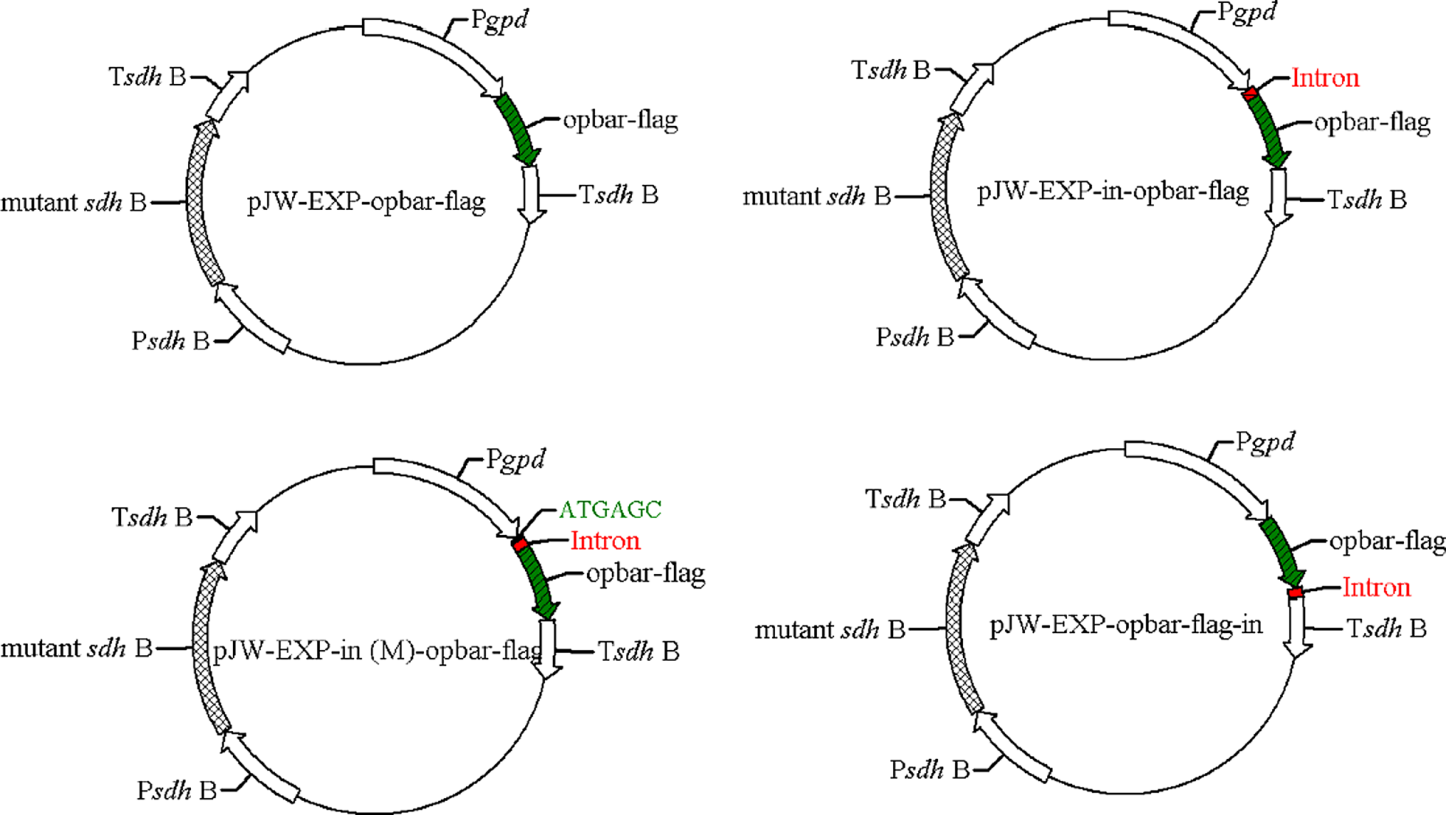
Structure of opbar-flag plasmids used for *G. lucidum* transformation. See “Methods” for details on plasmid construction

**Fig. 2:**
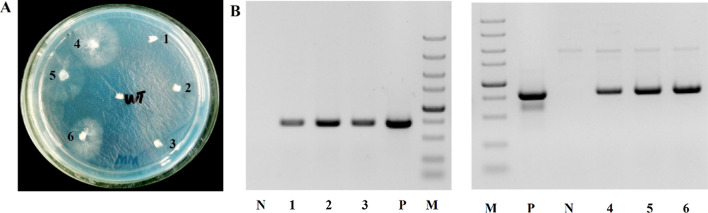
Selection of phosphinothrin-resistant transformants on a selective CYM plate. **A** Transformants on a selective CYM plate. 1, 2, 3: the strain transformed with pJW-EXP-opbar-flag; 4, 5, 6: the strain transformed with pJW-EXP-in-opbar-flag. **B** Identificaton of *G. lucidum* transformants by PCR. Lane M, DNA marker DL5000; Lane P, pJW-EXP-in-opbar-flag as positive control; Lane N, negative control; Lane 1, 2, 3, the transformants pJW-EXP-opbar-flag; Lane 4, 5, 6, the transformant pJW-EXP-in-opbar-flag

Our previous study has shown that the insertion of the *gpd* fragment containing the first exon (6 bp), the first intron (67 bp), and part of the second exon (3 bp) at the 5′ end of the *bar* and *gfp* exerts a significant influence on protein expression. To investigate the effect of the endogenous *gpd* intron 1 on protein expression in *G. lucidum*, we constructed plasmids in which the heterologous codon-optimized phosphinothricin-resistance gene (*opbar*)-*flag* was regulated by the endogenous *gpd* promoter and the succinate dehydrogenase gene (*sdh*) terminator of *G. lucidum* (pJW-EXP-opbar-flag) (Fig. 1). Alternatively, the *opbar*-*flag* was cloned in similar plasmids that contained the *gpd* intron 1 directly upstream of the start codon (pJW-EXP-in-opbar-flag), 6 bp downstream of the translation start site of *bar* (pJW-EXP-in (M)-opbar-flag), and directly downstream of the stop codon (pJW-EXP-opbar-flag-in) (Fig. 1). These plasmids also contained a carboxin-resistance cassette, allowing selection of transformants on CYM plates with carboxin. After *G. lucidum* protoplasts were transformed with intron-containing and intronless opbar-flag plasmids (pJW-EXP-opbar-flag), we obtained carboxin-resistant colonies with all used plasmids. However, when these carboxin-resistance transformants were re-screened on CYM plates including phosphinothrin, no phosphinothrin-resistant colonies were obtained with plasmid pJW-EXP-opbar-flag. The phosphinotrhrin-resistance transformants were obtained with intron-containing plasmids pJW-EXP-in-opbar-flag, pJW-EXP-in (M)-opbar-flag, and pJW-EXP-opbar-flag-in (Fig. 2A, Additional file 1: Figs. S1A and S2A). PCR analysis showed that the *opbar-flag* has integrated into the genomes of transformants pJW-EXP-opbar-flag, transformants pJW-EXP-in-opbar-flag, pJW-EXP-opbar-in (M)-flag, and pJW-EXP-opbar-flag-in (Fig. 2B, Additional file 1: Figs. S1B and S2B). These results indicated that the introduction of the endogenous *gpd* intron 1 is important for efficient expression of heterologous *bar* in *G. lucidum*. Moreover, this effect was independent of the position of the *gpd* intron 1. Positive effects of intron on transgene expression have also been observed in other basidiomycetes such as *Agaricus bisporus*, *C. cinereus*, *Phanerochaete chrysosporium*, *S. commune*, *C. passeckerianus* and *Trichoderma viride* [20–24]. Enhancement of gene expression by introns may play a role in those organisms that generally possess introns in their genes [22].

### Enhancement is associated with increased levels of mRNA

**Fig. 3:**
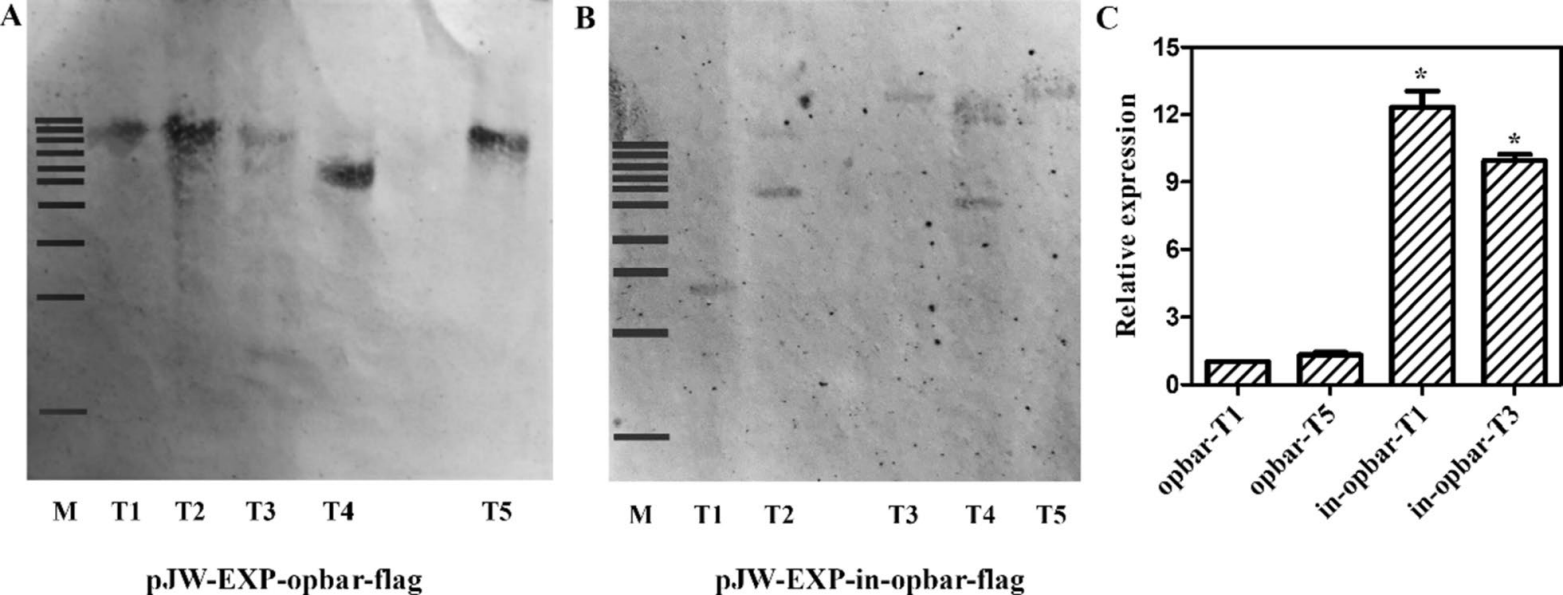
Detection of copies of *opbar* and transcriptional levels of *opbar* in different *G. lucidum* transformants. **A** pJW-EXP-opbar-flag transformants. **B** pJW-EXP-in-opbar-flag transformants. **C** The relative expression levels of *opbar* in transformants opbar-T1, opbar-T5, in-opbar-T1, and in-opbar-T3. Lane M, DNA marker 1 kb ladder

The transformant pJW-EXP-in-opbar-flag was chosen with the tranformant pJW-EXP-opbar-flag to study how the *gpd* intron 1 affects gene transcription and protein expression. The presence of opbar-flag in the genome of transformants pJW-EXP-opbar-flag and pJW-EXP-in-opbar-flag was further determined by molecular hybridization. The genome of transformants pJW-EXP-opbar-flag and pJW-EXP-in-opbar-flag were digested with NheI and probed with an *opbar* fragment (Fig. 3A, B). Southern blot analysis verified that these transformants had insertions of *opbar*, with single or multiple copies in their genomes. Single-copy integration of opbar-flag event was detected in the genomes of transformants pJW-EXP-opbar-flag T1, T4, and T5, and pJW-EXP-in-opbar-flag T1, T3, and T5. To minimize copy-number effect, the transcription level of *opbar* was analyzed in transformants pJW-EXP-opbar-flag T1 and T5, and pJW-EXP-in-opbar-flag T1 and T3 carrying a single-copy *opbar* by real-time qRT-PCR analysis. The results showed high transcription levels in transformants pJW-EXP-in-opbar-flag T1 and T3 bearing *gpd* intron 1 in *opbar* and low levels for transformants pJW-EXP-opbar-flag T1, and T5 containing *opbar* without *gpd* intron 1. No significant transcription difference was observed between pJW-EXP-in-opbar-flag T1 and T5. The transcription level of *opbar* in transformants pJW-EXP-in-opbar-flag T1 and T3 was, respectively, 12.3- and 10.1-fold higher than in transformant pJW-EXP-in-opbar-flag T1 (Fig. 3C). Our results indicated that *gpd* intron 1 increased the transcription level of heterologous *opbar* in *G. lucidum*. Previous studies have suggested that introns increased the level of mRNA possibly by enhancing maturation and stability of transcripts [25]. The difference in transcription level between pJW-EXP-in-opbar-flag T1 and T3 might be relevant to integration position effects [26].

### The effect of the *gpd* intron 1 on protein yield of heterologous *opbar*

**Fig. 4:**
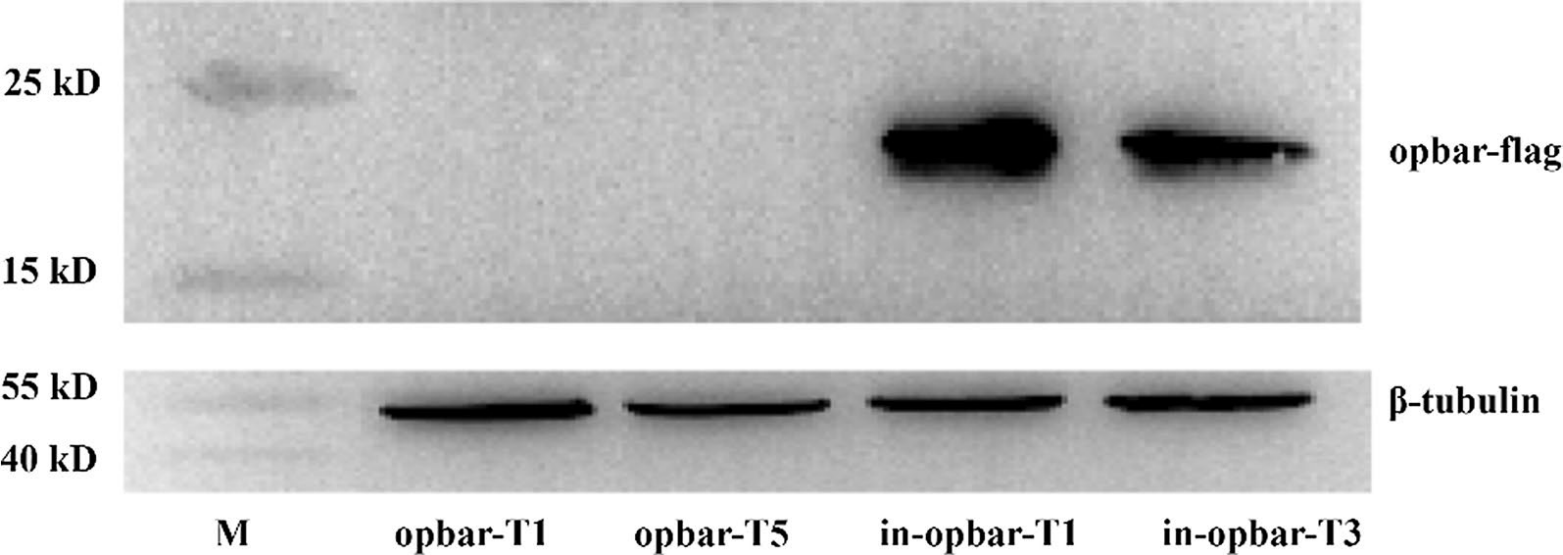
Analysis of opbar-flag amount by SDS-PAGE in transformants opbar-T1, opbar-T5, in-opbar-T1, and in-opbar-T3. Lane M, protein marker

Proteins isolated from transformants pJW-EXP-opbar-flag T1, and T5, and pJW-EXP-in-opbar-flag T1 and T3 were analyzed by western blotting (Fig. 4). A protein of ≈ 22 kDa was detectable in the transformants pJW-EXP-in-opbar-flag T1 and T3. However, it was not observed in transformants pJW-EXP-opbar-flag T1 and T5 containing *opbar* without the *gpd* intron 1. The band at 22 kDa that was recognized by flag-specific antibodies was the opbar-flag protein, as the molecular weight was similar to the predicted value. As shown in Fig. 4, the amount of opbar-flag protein also increased with the introduction of *gpd* intron 1. These results coincided with those of qRT-PCR analysis and phosphinothrin-resistance screening. G*pd* intron 1 enhanced protein expression both at the RNA and protein levels. Opbar protein levels did not correlate with *opbar* mRNA levels in the intronless *G. lucidum* transformants, indicating that *gpd* intron 1 may also enhance the efficiency of translation besides increasing mRNA content by affecting transcription and stability of mRNA [27, 28]. The detailed mechanism of enhancement of introns on the translation of heterologous genes requires further investigation.

### Introduction of *gpd* intron1 enhances β-glucuronidase (GUS) expression

**Fig. 5:**
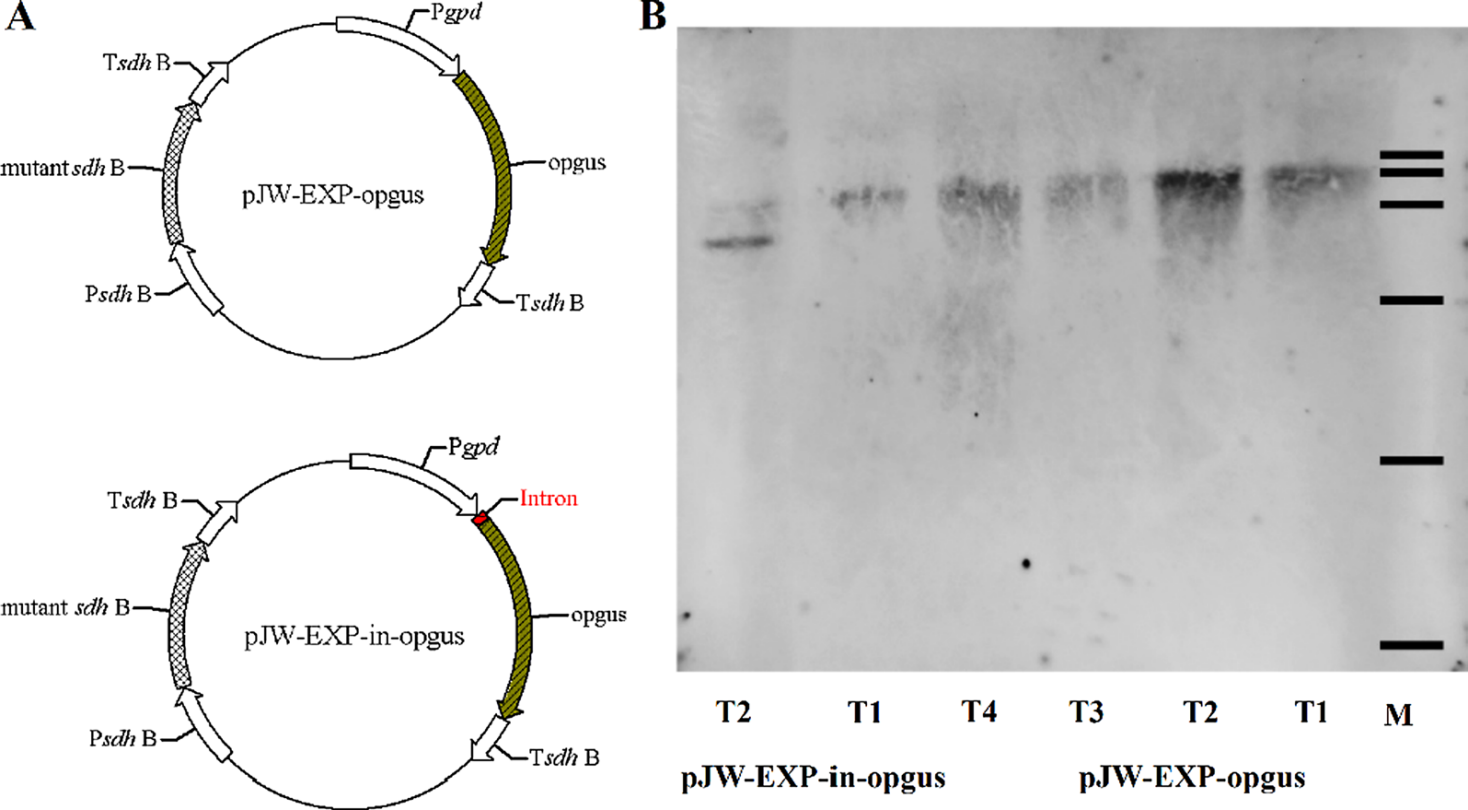
Detection of copies of *opgus* in different *G. lucidum* transformants. **A** Structure of opgus plasmids. **B** Genome southern blot analysis of pJW-EXP-opgus transformant T1, T2, T3, and T4, and pJW-EXP-in-opgus transformants T1 and T2. Lane M, DNA marker DL 15,000

**Fig. 6:**
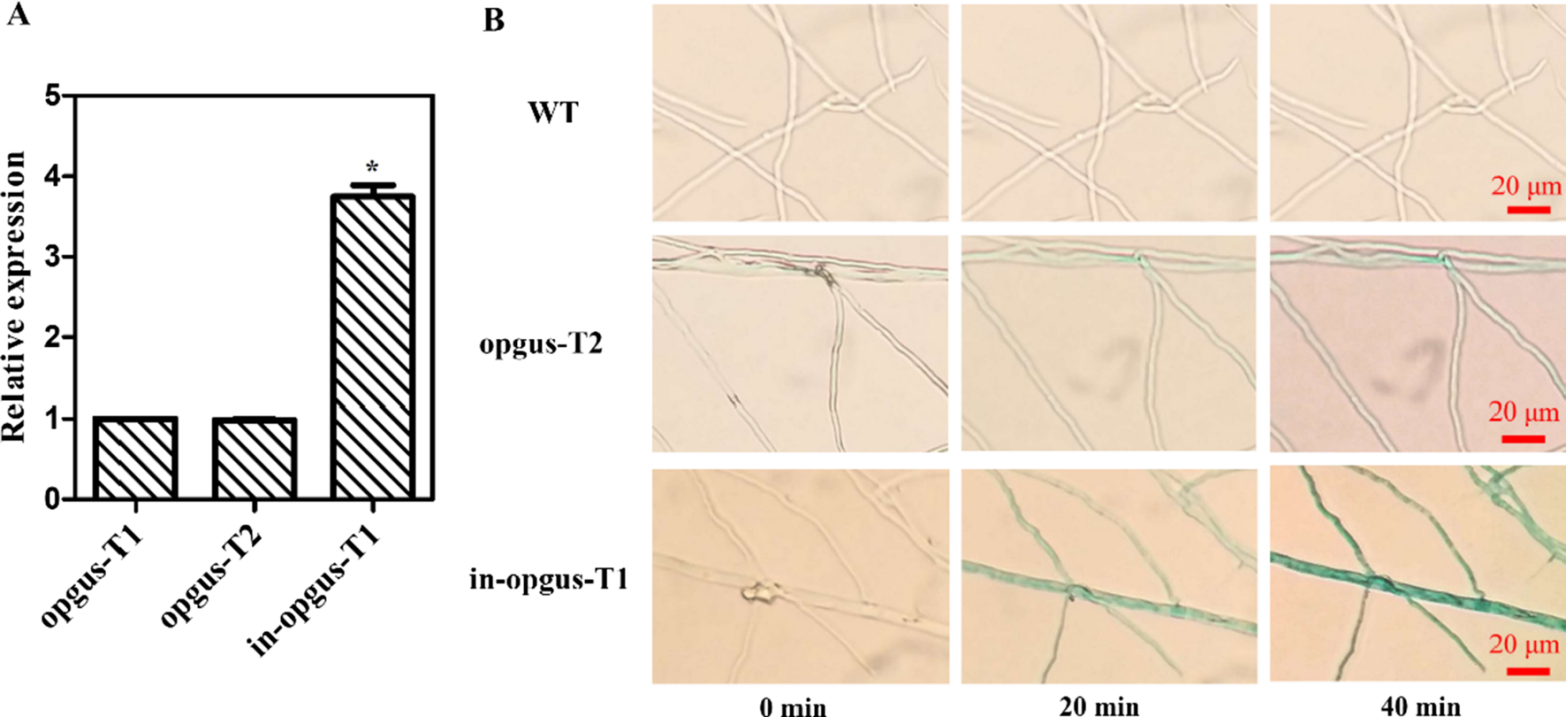
The relative expression levels of *opbar* (**A**) in and histochemical staining (**B**) of transformants opgus-T1, opgus-T2, and in-opgus-T1

To confirm the positive effect of *gpd* intron 1 on the expression of heterologous genes, we also constructed plasmids pJW-EXP-opgus and pJW-EXP-in-opgus (Fig. 5A) and transformed these into *Ganoderma* protoplasts. These plasmids contain a carboxin-resistant cassette for transformation selection and the full codon-optimized *gus* (*opgus*) cassette (pJW-EXP-opgus) or the *intron*-*opgus* cassette. Carboxin-resistant colonies were screened for transgenes by genome PCR (Additional file 1: Fig. S3) and subsequently were further analyzed by Southern blotting. The results showed that *opgus* and *intro-opgu*s had integrated into the genome of the recipient as single copies in transformants pJW-EXP-opgus T1, T2, T3, and T4, and transformants pJW-EXP-in-opgus T1 (Fig. 5B). Real-time qRT-PCR analysis showed that the transcription level of *opgus* in transformant pJW-EXP-in-opgus T1 was 3.8-fold higher than that in transformants pJW-EXP-opgus T1 and T2 without *gpd* intron 1 (Fig. 6A). The increase in transcription level relative to the intronless control ranged from 3.8-fold for *opgu*s to 12.3-fold for *opbar*, which may be dependent on the cDNA sequence and the transcription level of the intronless heterologous gene [27, 29]. Histochemical staining analysis was also performed to detect expression of *opgus* in wild-type (WT), and transformants pJW-EXP-opgus T2 and pJW-EXP-in-opgus T1. Figure 6B shows that GUS activity was observed in the mycelia of transformants pJW-EXP-opgus T2 and pJW-EXP-in-opgus T1, while no blue staining was detected in the WT mycelia. Moreover, GUS activity in transformant pJW-EXP-in-opgus T1 was higher than that in transformant pJW-EXP-opgus T2. Again, our results showed that *gpd* intron 1 enhanced the *opgus* transcription and GUS enzyme activity in *G. lucidum*. It may thus be possible to efficiently express other heterologous genes in *Ganoderma* by introducing the *gpd* intron 1.

## Conclusions

This work demonstrated the importance of introducing *gpd* intron 1 for the efficient expression of *opba*r and *gus* in *G. lucidum*. The presence of *gpd* intron 1 in heterologous genes enhances mRNA accumulation and protein expression in basidiomycete *Ganoderma*. The developed method can be applicable to upregulate the expression of other heterologous genes in *Ganoderma*.

## Methods

### Strains and media

Monokaryotic *G. lucidum* 5.616-1 strain [30] was cultured in CYM plates (10 g/L maltose, 20 g/L glucose, 2 g/L tryptone, 2 g/L yeast extract, 0.5 g/L MgSO_4_, 4.6 g/L KH_2_PO_4_ and 10 g/L agar) and in transformations. *Escherichia coli* strain DH5α was used in the construction and transformation of plasmids. The mycelia of *G. lucidum* were cultured in fermentation medium (35 g/L glucose, 1 g/L KH_2_PO_4_·H_2_O, 0.5 g/L MgSO_4_·7H_2_O, 5 g/L peptone, 2.5 g/L yeast extract, and 0.05 g/L vitamin B1, pH 5.5) in the dark at 30 °C [31, 32].

### Plasmid construction

The codon optimized phosphinothricin-resistance gene (opbar)-flag, *gpd* gene intron 1 (in)-opbar-flag, in(M)-opbar-flag, *opbar*-flag-in, opgus, and in-opgus (Additional file 1) were synthesized by Sangon Co., Ltd. (Shanghai, China). These genes were ligated into pUC57 (Sangon) to produce plasmids, pUC57-opbar-flag, pUC57-in-opbar-flag, pUC57-in (M)-opbar-flag, pUC57-opbar-flag-in, pUC57-opgus, and pUC57-in-opgus, respectively.

The opbar-flag, in-opbar-flag, in(M)-opbar-flag, and opbar-flag-in genes were PCR amplified from plasmids pUC57-opbar-flag, pUC57-in-opbar-flag, pUC57-in (M)-opbar-flag, and pUC57-opbar-flag-in using primers opbar-flag-F/opbar-flag-R, in-opbar-flag F/opbar-flag-R, opbar-flag F2/opbar-flag-R, and opbar-flag-F/opbar-flag R2 (Additional file 1: Table S1), respectively. These PCR products were fused into plasmid pJW-EXP [13] that were digested with NheI and SmaI using the ClonExpress MultiS One Step Cloning Kit (Vazyme, Nanjing), yielding plasmid pJW-EXP-opbar-flag, pJW-EXP-in-opbar-flag, pJW-EXP-in(M)-opbar-flag, and pJW-EXP-opbar-flag-in, respectively.

The opgus and in-opgus genes were PCR amplified from plasmids pUC57-opgus and pUC57-in-opgus using primers opgus F/opgus R and in-opgus F/opgus R (Additional file 1: Table S1), respectively. The obtained products were fused into plasmid pJW-EXP that was digested with NheI and SmaI using the ClonExpress MultiS One Step Cloning Kit (Vazyme, Nanjing) to generate plasmids pJW-EXP-opgus and pJW-EXP-in-opgus, respectively.

### Genetic transformation of *G. lucidum* and selection of transformants

Transformation of *G. lucidum* protoplasts was performed according to our previous studies [13, 33]. The suspensions of *G. lucidum* protoplasts and plsmids in PTC buffer (50 mM CaCl_2_, 10 mM Tris-HCl buffer (pH 7.5), and 60% w/v PEG 4000) were plated onto CYM selective plates containing 0.6 M mannitol and 2 µg/L carboxine. After 14 days of incubation at 30 °C, carboxin-resistant transformants were screened for phosphinothricin-resistance protein and GUS expression. The transformants expressing *opbar* were screened on CYM plates with 150 mg/L phosphinothricin. The genome of these transformants was extracted for PCR amplification of the fused fragment of *gpd* promoter and *opbar* using primers gpd-id-F/opbar-id-R. The transformants expressing *opgus* were identified by PCR amplification of the fused fragment of *gpd* promoter and *opgus* using primers gpd-id-F/opgus-R.

### Nucleic acid isolation

*Ganoderma lucidum* mycelia were harvested, frozen, and powdered in liquid nitrogen. Genomic DNA was isolated employing the cetyltrimethylammonium bromide (CTAB) method. Total RNA was isolated using TRIzol reagent (Invitrogen, Carlsbad, CA, USA) according to the manufacturer’s protocols and treated with RNase-free DNase I before use.

### Southern blots

DNA samples from *G. lucidum* transformants were digested with NheI and separated on a 0.7% agarose gel. The *opbar* (0.54 kb) and *opgus* (0.95 kb) fragment amplified from plasmids pJW-EXP-opbar-flag and pJW-EXP-opgus with primers bar probe F/bar probe F and gus probe F/gus probe R were used as probes. Southern blot analysis was conducted under conditions recommended for the digoxigenin (DIG) hybridization system by Mylab™ (Beijing, China).

### Quantitative real-time (qRT)-PCR analysis

Approximately 1 mg total RNA was used as template, and reverse transcription was performed using the PrimeScript™ RT reagent Kit (Takara, China) following the manufacturer’s instructions. The transcription levels of *opbar* and *opgus* were determined by qRT-PCR as previously described [6, 16]. The 18S-rRNA transcript was used as internal control to normalize relative transcription levels. For *opbar* and *opgus*, the transcription level in opbar-flag and opgus strains was set to 1.0, and the relative transcription levels in other strains were presented as fold changes relative to the reference level. The primers qRT-bar-F/qRT-bar-R, qRT-gus-F/qRT-gus-R, and qRT-18S-F/qRT-18S-R (Additional file 1: Table S1) were used for amplification of the *opbar*, *opgus*, and 18S rRNA genes, respectively.

### Western blot analysis

*Ganoderma lucidum* mycelia were washed and homogenized in 1 mL of lysis buffer (25 mM Tris base (pH 7.4), 200 mM glycine, 5 mM EDTA, 1 mM phenylmethyl sulfonyl fluoride, and 1 mM 2-mercaptoethanol). The homogenates were centrifuged at 12,000×*g* for 20 min at 4 °C, then protein concentration in the supernatants was determined using the Bradford method. For western bolt analysis, 30 µg of protein was separated on a 12% SDS-polyacrylamide gel and electro-transferred to a polyvinylidene difluoride membrane in TBST buffer [10 mM Tris/HCl (pH 7.4), 100 mM NaCl, 0.1% Tween 20] for 1 h. Membranes were blocked with non-fat milk dissolved in TBSF buffer at room temperature for 2 h, followed by incubation with primary antibodies (anti-Flag, PROTEINTECH, Cat No. 20543-1-AP at 1:5000 dilution; anti-tubulin rabbit polyclonal antibody, BBI, Cat No. D110015 at 1:5000 dilution). Membranes were washed with TBSF and incubated with an HRP-conjugated goat anti-rabbit IgG (BBI, Cat No. D110058 at 1:5000 dilution). The blots were washed with TBSF buffer again, and the bands were visualized using an enhanced chemiluminescene method.

### GUS activity assay

*Ganoderma lucidum* mycelia scraped from CYM plate were stained with GUS detection buffer [0.1 M sodium phosphate (pH 7.0), 0.5 mg/mL X-Gluc, 0.05 mM K_3_ (Fe[CN]_6_), 0.05 mM K_4_(Fe[CN]_6_), and 0.5% Triton X-100] on a glass slide for 20 and 40 min, respectively. After washing with PB buffer (10 mM sodium phosphate, pH 7.0), the mycelia were observed under a Nikon Coolpix 900 camera.

### Statistical analysis

Data were generated in three independent sample measurements, and all data are presented as the mean ± standard deviation. The results were considered significant for *p* values < 0.05 in a two-tailed analysis.

## Supplementary Information

**Additional file 1.** Data S1. The synthesized gene sequence. **Fig. S1**. Selection of phosphinothrin-resistant transformants on a selective CYM plate. (A) Transformants on a selective CYM plate. 1, 2, 3, 4: Strains transformed with pJW-EXP-in (M)-opbar-flag. (B) Identificaton of *G. lucidum* transformants by PCR. **Fig. S2**. Selection of phosphinothrin-resistant transformants on a selective CYM plate. (A) Transformants on a selective CYM plate. 1, 2, 3, 4: Strains transformed with pJW-EXP-opbar-flag-in. (B) Identificaton of *G. lucidum* transformants by PCR. **Fig. S3**. Identificaton of *G. lucidum* transformants with plasmid pJW-EXP-opgus (A) and pJW-EXP-in-opgus (B) by PCR. **Table S1**. Oligonucleotides used in this study.
