# Supplementary material for: Efficient expression of heterologous genes by the introduction of the endogenous glyceraldehyde-3-phosphate dehydrogenase gene intron 1 in Ganoderma lucidum
Source: Microb Cell Fact. 2021 Aug 21;20:164. doi: 10.1186/s12934-021-01654-8 (PMC8379801; doi:10.1186/s12934-021-01654-8)
Supplement: Supplementary file 1 — Additional file 1. Data S1. The synthesized gene sequence. Fig. S1. Selection of phosphinothrin-resistant transformants on a selective CYM plate. (A) Transformants on a selective CYM plate. 1, 2, 3, 4: Strains transformed with pJW-EXP-in (M)-opbar-flag. (B) Identificaton of G. lucidum transformants by PCR. Fig. S2. Selection of phosphinothrin-resistant transformants on a selective CYM plate. (A) Transformants on a selective CYM plate. 1, 2, 3, 4: Strains transformed with pJW-EXP-opbar-flag-in. (B) Identificaton of G. lucidum transformants by PCR. Fig. S3. Identificaton of G. lucidum transformants with plasmid pJW-EXP-opgus (A) and pJW-EXP-in-opgus (B) by PCR. Table S1. Oligonucleotides used in this study. [file 12934_2021_1654_MOESM1_ESM.docx]

**Additional file 1**

**Data S1-The synthesized gene sequence**

opbar-flag:

ATGAGCCCAGAGCGTCGTCCTGCTGACATCCGCCGTGCTACCGAGGCTGACATGCCAGCGGTTTGCACCATCGTCAACCACTACATCGAGACGAGCACGGTCAACTTCCGCACCGAGCCCCAGGAGCCTCAGGAGTGGACGGATGACCTCGTCCGTCTGCGGGAGCGCTACCCCTGGCTCGTCGCCGAGGTCGACGGAGAGGTCGCCGGTATCGCTTACGCGGGTCCTTGGAAGGCCCGCAACGCCTACGATTGGACGGCCGAGTCTACCGTGTACGTCTCCCCCCGCCATCAACGGACCGGCCTTGGTTCCACCCTCTATACTCACCTGCTGAAGTCCCTCGAGGCCCAGGGCTTCAAGTCGGTGGTCGCGGTCATTGGGCTCCCCAACGACCCTTCGGTGCGCATGCACGAAGCGCTCGGCTATGCCCCCCGCGGCATGCTTCGCGCGGCCGGCTTCAAACACGGGAATTGGCACGACGTGGGCTTCTGGCAGCTTGACTTCTCGCTCCCCGTCCCGCCGCGCCCGGTCCTCCCCGTTACTGAAATTGACTACAAGGATGACGACGACAAGTGA

In-opbar-flag:

GTGAGTCCTGCATCCCCATCGTGCACCGTATTCACCTCATCGTTTGGCCCCCTTCTCACAGGTCAAGATGAGCCCAGAGCGTCGTCCTGCTGACATCCGCCGTGCTACCGAGGCTGACATGCCAGCGGTTTGCACCATCGTCAACCACTACATCGAGACGAGCACGGTCAACTTCCGCACCGAGCCCCAGGAGCCTCAGGAGTGGACGGATGACCTCGTCCGTCTGCGGGAGCGCTACCCCTGGCTCGTCGCCGAGGTCGACGGAGAGGTCGCCGGTATCGCTTACGCGGGTCCTTGGAAGGCCCGCAACGCCTACGATTGGACGGCCGAGTCTACCGTGTACGTCTCCCCCCGCCATCAACGGACCGGCCTTGGTTCCACCCTCTATACTCACCTGCTGAAGTCCCTCGAGGCCCAGGGCTTCAAGTCGGTGGTCGCGGTCATTGGGCTCCCCAACGACCCTTCGGTGCGCATGCACGAAGCGCTCGGCTATGCCCCCCGCGGCATGCTTCGCGCGGCCGGCTTCAAACACGGGAATTGGCACGACGTGGGCTTCTGGCAGCTTGACTTCTCGCTCCCCGTCCCGCCGCGCCCGGTCCTCCCCGTTACTGAAATTGACTACAAGGATGACGACGACAAGTGA

In (M)-op*bar*-flag:

ATGAGCGTGAGTCCTGCATCCCCATCGTGCACCGTATTCACCTCATCGTTTGGCCCCCTTCTCACAGGTCAAGCCAGAGCGTCGTCCTGCTGACATCCGCCGTGCTACCGAGGCTGACATGCCAGCGGTTTGCACCATCGTCAACCACTACATCGAGACGAGCACGGTCAACTTCCGCACCGAGCCCCAGGAGCCTCAGGAGTGGACGGATGACCTCGTCCGTCTGCGGGAGCGCTACCCCTGGCTCGTCGCCGAGGTCGACGGAGAGGTCGCCGGTATCGCTTACGCGGGTCCTTGGAAGGCCCGCAACGCCTACGATTGGACGGCCGAGTCTACCGTGTACGTCTCCCCCCGCCATCAACGGACCGGCCTTGGTTCCACCCTCTATACTCACCTGCTGAAGTCCCTCGAGGCCCAGGGCTTCAAGTCGGTGGTCGCGGTCATTGGGCTCCCCAACGACCCTTCGGTGCGCATGCACGAAGCGCTCGGCTATGCCCCCCGCGGCATGCTTCGCGCGGCCGGCTTCAAACACGGGAATTGGCACGACGTGGGCTTCTGGCAGCTTGACTTCTCGCTCCCCGTCCCGCCGCGCCCGGTCCTCCCCGTTACTGAAATTGACTACAAGGATGACGACGACAAGTGA

op*bar*-flag-in:

ATGAGCCCAGAGCGTCGTCCTGCTGACATCCGCCGTGCTACCGAGGCTGACATGCCAGCGGTTTGCACCATCGTCAACCACTACATCGAGACGAGCACGGTCAACTTCCGCACCGAGCCCCAGGAGCCTCAGGAGTGGACGGATGACCTCGTCCGTCTGCGGGAGCGCTACCCCTGGCTCGTCGCCGAGGTCGACGGAGAGGTCGCCGGTATCGCTTACGCGGGTCCTTGGAAGGCCCGCAACGCCTACGATTGGACGGCCGAGTCTACCGTGTACGTCTCCCCCCGCCATCAACGGACCGGCCTTGGTTCCACCCTCTATACTCACCTGCTGAAGTCCCTCGAGGCCCAGGGCTTCAAGTCGGTGGTCGCGGTCATTGGGCTCCCCAACGACCCTTCGGTGCGCATGCACGAAGCGCTCGGCTATGCCCCCCGCGGCATGCTTCGCGCGGCCGGCTTCAAACACGGGAATTGGCACGACGTGGGCTTCTGGCAGCTTGACTTCTCGCTCCCCGTCCCGCCGCGCCCGGTCCTCCCCGTTACTGAAATTGACTACAAGGATGACGACGACAAGTGAGTGAGTCCTGCATCCCCATCGTGCACCGTATTCACCTCATCGTTTGGCCCCCTTCTCACAGGTCAAG

opgus:

ATGCTCCGCCCCGTCGAGACCCCCACGCGCGAGATCAAGAAGCTCGACGGCCTCTGGGCCTTCTCGCTCGACCGCGAGAACTGCGGCATCGACCAGCGCTGGTGGGAGAGCGCCCTCCAGGAGTCCCGCGCCATCGCGGTCCCGGGCTCCTTCAACGACCAGTTCGCCGACGCGGACATCCGCAACTACGCCGGCAACGTCTGGTACCAGCGCGAGGTCTTCATCCCCAAGGGCTGGGCCGGCCAGCGCATCGTCCTGCGCTTCGACGCCGTCACGCACTACGGCAAGGTCTGGGTCAACAACCAGGAGGTCATGGAGCACCAGGGCGGCTACACCCCTTTCGAGGCGGACGTGACCCCCTACGTCATCGCCGGCAAGTCGGTCCGCATCACCGTCTGCGTCAACAACGAGCTCAACTGGCAGACCATTCCGCCCGGCATGGTCATCACCGACGAGAACGGCAAGAAGAAGCAGTCGTACTTCCATGACTTCTTCAACTACGCCGGCATCCACCGTTCCGTCATGCTCTACACCACCCCCAACACCTGGGTCGACGACATCACCGTCGTGACCCACGTCGCCCAGGACTGCAACCACGCCTCCGTCGACTGGCAGGTGGTCGCCAACGGCGACGTCTCGGTCGAGCTCCGCGACGCGGACCAGCAGGTCGTGGCCACCGGCCAGGGCACGTCCGGCACGCTTCAGGTCGTGAACCCCCACCTCTGGCAGCCAGGCGAGGGCTACCTCTACGAGCTCTGCGTCACCGCCAAGAGCCAGACCGAGTGCGACATCTACCCGCTCCGCGTGGGCATCCGCTCCGTCGCGGTGAAGGGCGAGCAGTTCCTCATCAACCATAAGCCTTTCTACTTCACCGGGTTCGGCCGCCACGAGGACGCCGACCTCCGCGGCAAGGGGTTCGACAACGTCCTCATGGTGCACGACCACGCCCTCATGGACTGGATCGGCGCCAACTCCTACCGCACCAGCCATTACCCTTACGCCGAGGAGATGCTCGACTGGGCCGACGAGCACGGCATCGTCGTCATCGACGAGACCGCCGCGGTGGGTTTCAACCTGAGCCTCGGCATCGGCTTCGAGGCGGGCAACAAGCCCAAGGAGCTCTACAGCGAGGAGGCCGTCAACGGCGAGACGCAGCAGGCCCACCTCCAGGCCATCAAGGAGCTGATCGCGCGTGACAAGAACCACCCCTCGGTCGTCATGTGGTCCATCGCGAACGAGCCCGACACCCGCCCCCAGGGTGCGCGCGAGTACTTCGCCCCCCTCGCCGAGGCGACCCGCAAGCTGGACCCCACCCGCCCCATCACCTGCGTCAACGTCATGTTCTGTGACGCTCACACCGACACCATCTCGGACCTCTTCGACGTCCTGTGCCTCAACCGCTACTACGGGTGGTACGTCCAGTCCGGCGACCTCGAGACCGCCGAGAAGGTCCTCGAGAAGGAGCTCCTGGCGTGGCAGGAGAAGCTCCACCAGCCCATCATCATCACGGAGTACGGCGTCGACACGCTCGCCGGCCTCCACTCGATGTACACCGACATGTGGTCGGAGGAGTACCAGTGCGCCTGGCTCGACATGTACCACCGTGTCTTCGACCGCGTCAGCGCCGTCGTCGGCGAGCAGGTCTGGAACTTCGCGGACTTCGCCACCTCGCAAGGCATTCTCCGCGTCGGCGGCAACAAGAAGGGCATCTTCACCCGCGACCGCAAGCCTAAGTCCGCGGCCTTCCTCCTCCAGAAGCGCTGGACGGGCATGAACTTCGGCGAGAAGCCCCAGCAGGGCGGCAAGCAGTGA

In-opgus:

GTGAGTCCTGCATCCCCATCGTGCACCGTATTCACCTCATCGTTTGGCCCCCTTCTCACAGGTCAAGATGCTCCGCCCCGTCGAGACCCCCACGCGCGAGATCAAGAAGCTCGACGGCCTCTGGGCCTTCTCGCTCGACCGCGAGAACTGCGGCATCGACCAGCGCTGGTGGGAGAGCGCCCTCCAGGAGTCCCGCGCCATCGCGGTCCCGGGCTCCTTCAACGACCAGTTCGCCGACGCGGACATCCGCAACTACGCCGGCAACGTCTGGTACCAGCGCGAGGTCTTCATCCCCAAGGGCTGGGCCGGCCAGCGCATCGTCCTGCGCTTCGACGCCGTCACGCACTACGGCAAGGTCTGGGTCAACAACCAGGAGGTCATGGAGCACCAGGGCGGCTACACCCCTTTCGAGGCGGACGTGACCCCCTACGTCATCGCCGGCAAGTCGGTCCGCATCACCGTCTGCGTCAACAACGAGCTCAACTGGCAGACCATTCCGCCCGGCATGGTCATCACCGACGAGAACGGCAAGAAGAAGCAGTCGTACTTCCATGACTTCTTCAACTACGCCGGCATCCACCGTTCCGTCATGCTCTACACCACCCCCAACACCTGGGTCGACGACATCACCGTCGTGACCCACGTCGCCCAGGACTGCAACCACGCCTCCGTCGACTGGCAGGTGGTCGCCAACGGCGACGTCTCGGTCGAGCTCCGCGACGCGGACCAGCAGGTCGTGGCCACCGGCCAGGGCACGTCCGGCACGCTTCAGGTCGTGAACCCCCACCTCTGGCAGCCAGGCGAGGGCTACCTCTACGAGCTCTGCGTCACCGCCAAGAGCCAGACCGAGTGCGACATCTACCCGCTCCGCGTGGGCATCCGCTCCGTCGCGGTGAAGGGCGAGCAGTTCCTCATCAACCATAAGCCTTTCTACTTCACCGGGTTCGGCCGCCACGAGGACGCCGACCTCCGCGGCAAGGGGTTCGACAACGTCCTCATGGTGCACGACCACGCCCTCATGGACTGGATCGGCGCCAACTCCTACCGCACCAGCCATTACCCTTACGCCGAGGAGATGCTCGACTGGGCCGACGAGCACGGCATCGTCGTCATCGACGAGACCGCCGCGGTGGGTTTCAACCTGAGCCTCGGCATCGGCTTCGAGGCGGGCAACAAGCCCAAGGAGCTCTACAGCGAGGAGGCCGTCAACGGCGAGACGCAGCAGGCCCACCTCCAGGCCATCAAGGAGCTGATCGCGCGTGACAAGAACCACCCCTCGGTCGTCATGTGGTCCATCGCGAACGAGCCCGACACCCGCCCCCAGGGTGCGCGCGAGTACTTCGCCCCCCTCGCCGAGGCGACCCGCAAGCTGGACCCCACCCGCCCCATCACCTGCGTCAACGTCATGTTCTGTGACGCTCACACCGACACCATCTCGGACCTCTTCGACGTCCTGTGCCTCAACCGCTACTACGGGTGGTACGTCCAGTCCGGCGACCTCGAGACCGCCGAGAAGGTCCTCGAGAAGGAGCTCCTGGCGTGGCAGGAGAAGCTCCACCAGCCCATCATCATCACGGAGTACGGCGTCGACACGCTCGCCGGCCTCCACTCGATGTACACCGACATGTGGTCGGAGGAGTACCAGTGCGCCTGGCTCGACATGTACCACCGTGTCTTCGACCGCGTCAGCGCCGTCGTCGGCGAGCAGGTCTGGAACTTCGCGGACTTCGCCACCTCGCAAGGCATTCTCCGCGTCGGCGGCAACAAGAAGGGCATCTTCACCCGCGACCGCAAGCCTAAGTCCGCGGCCTTCCTCCTCCAGAAGCGCTGGACGGGCATGAACTTCGGCGAGAAGCCCCAGCAGGGCGGCAAGCAGTGA

The flag sequence are underlined. The *gpd* intron sequence was highlighted in red-color font.

**Fig. S1**

**
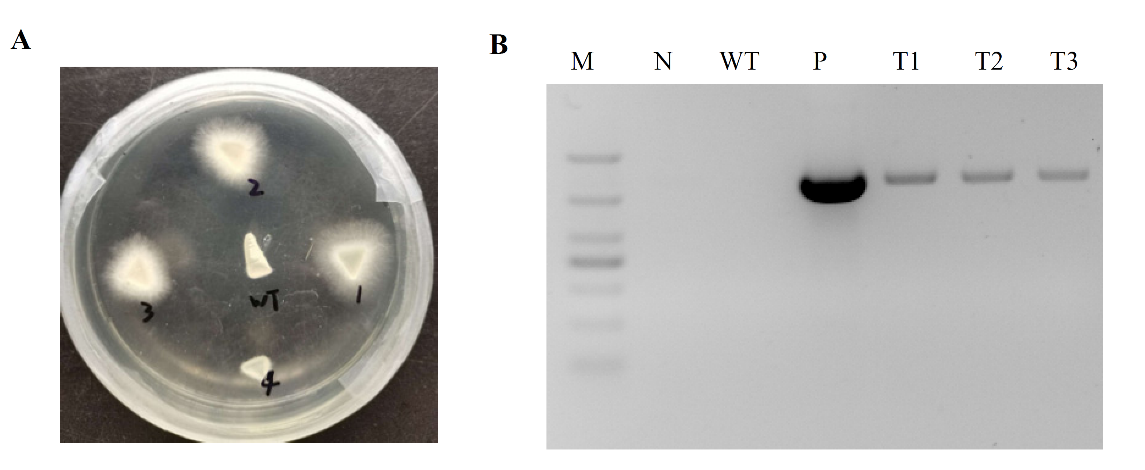
**

Fig. S1. Selection of phosphinothrin-resistant transformants on a selective CYM plate. (A) Transformants on a selective CYM plate. 1, 2, 3, 4: Strains transformed with pJW-EXP-in (M)-opbar-flag (B) Identificaton of *G. lucidum* transformants by PCR.

**Fig. S2**

**
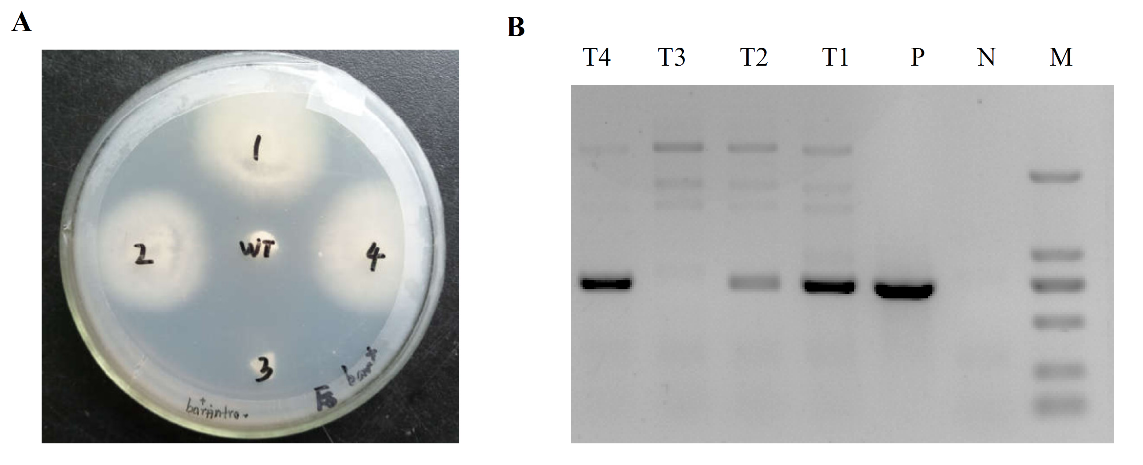
**

Fig. S1. Selection of phosphinothrin-resistant transformants on a selective CYM plate. (A) Transformants on a selective CYM plate. 1, 2, 3, 4: Strains transformed with pJW-EXP-opbar-flag-in. (B) Identificaton of *G. lucidum* transformants by PCR.

**Fig. S3**

**
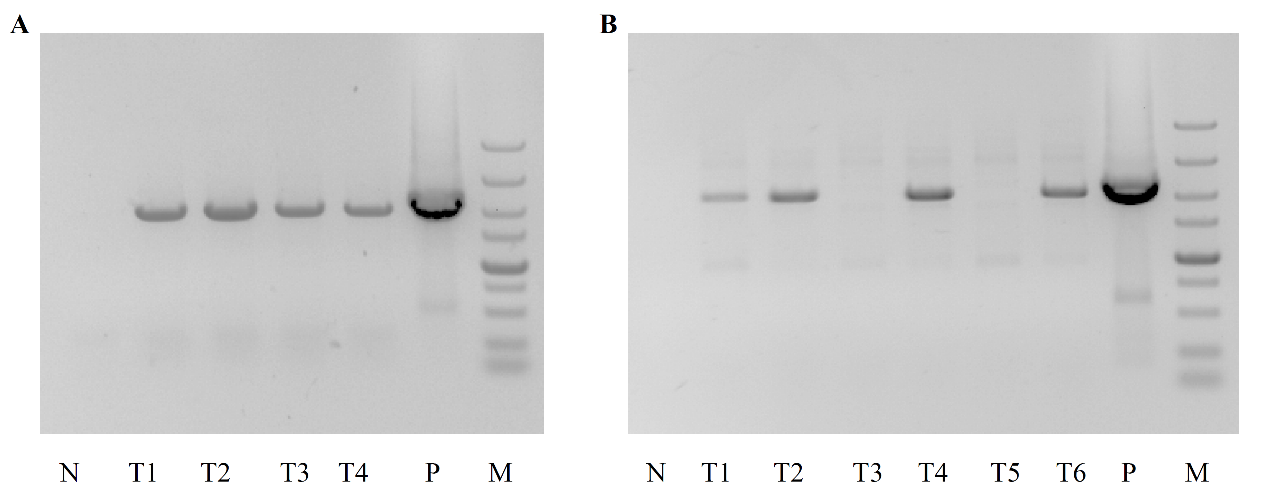
**

Identificaton of *G. lucidum* transformants with plasmid pJW-EXP-opgus (A) and pJW-EXP-in-opgus (B) by PCR.

**Table S1.** Oligonucleotides used in this study

| Target | Primers | Sequence (5’ 3’) |
| --- | --- | --- |
| Opbar-flag | Opbar-flag-F | TCATCCCCCTCTCAACATGAGCCCAGAGCGTCGT |
|  | Opbar-flag-R | CTCTGACCCGCTCATTCACTTGTCGTCGTCATCCTTG |
| In-opbar-flag | In-opbar-flag-F | TCATCCCCCTCTCAACGTGAGTCCTGCATCCCCAT |
|  | Opbar-flag-R | CTCTGACCCGCTCATTCACTTGTCGTCGTCATCCTTG |
| Opbar-in (M)-flag | Opbar-flag-F2 | TCATCCCCCTCTCAACATGAGCGTGAGTCCTGCATC |
|  | Opbar-flag-R | CTCTGACCCGCTCATTCACTTGTCGTCGTCATCCTTG |
| Opbar-flag-in | Opbar-flag-F | TCATCCCCCTCTCAACATGAGCCCAGAGCGTCGT |
|  | Opbar-flag-R2 | CTCTGACCCGCTCATCTTGACCTGTGAGAAGGGGG |
| Opgus | opgus-F | TCATCCCCCTCTCAACATGCTCCGCCCCGTC |
|  | opgus-R | CTCTGACCCGCTCATTCACTGCTTGCCGCCCT |
| In-opgus | In-opgus-F | TCATCCCCCTCTCAACGTGAGTCCTGCATCCCCAT |
|  | opgus-R | CTCTGACCCGCTCATTCACTGCTTGCCGCCCT |
| Gpd-opbar | Gpd-id-F | CGAGTGACGCAGGTGGTGAC |
|  | Opbar-id-R | ATTTCAGTAACGGGGAGGACC |
| Gpd-opgus | Gpd-id-F | CGAGTGACGCAGGTGGTGAC |
|  | Opgus-R | CTCTGACCCGCTCATTCACTGCTTGCCGCCCT |
| 18S-rRNA | qRT-18S-F | TATCGAGTTCTGACTGGGTTGT |
|  | qRT-18S-R | ATCCGTTGCTGAAAGTTGTAT |
| Opbar | qRT-opbar-F | GCACCATCGTCAACCACTACA |
|  | qRT-opbar-R | CCGTCCAATCGTAGGCGTT |
| Opgus | qRT-opgus-F | TCCTGTGCCTCAACCGCTA |
|  | qRT-opgus-R | CGCACTGGTACTCCTCCGAC |
| Opbar probe | Opbar probe-F | GAGCCCAGAGCGTCGTC |
|  | Opbar probe-R | TCACTTGTCGTCGTCATCCT |
| Opgus probe | Opgus probe-F | CGGCAAGAAGAAGCAGTCGT |
|  | Opgus probe-R | TAGCGGTTGAGGCACAGGA |
